# Granulin Secreted by the Food-Borne Liver Fluke *Opisthorchis viverrini* Promotes Angiogenesis in Human Endothelial Cells

**DOI:** 10.3389/fmed.2018.00030

**Published:** 2018-02-16

**Authors:** Brandon Haugen, Shannon E. Karinshak, Victoria H. Mann, Anastas Popratiloff, Alex Loukas, Paul J. Brindley, Michael J. Smout

**Affiliations:** ^1^Department of Microbiology, Immunology and Tropical Medicine, Research Center for Neglected Diseases of Poverty, School of Medicine & Health Sciences, George Washington University, Washington, DC, United States; ^2^Department of Biology, University of the District of Columbia, Washington, DC, United States; ^3^Nanofabrication and Imaging Center, Office of VP for Research, George Washington University, Washington, DC, United States; ^4^Centre for Biodiscovery and Molecular Development of Therapeutics, Australian Institute of Tropical Health and Medicine, James Cook University, Cairns, QLD, Australia

**Keywords:** granulin, parasite, angiogenesis, wound healing, liver cancer, human umbilical vein endothelial cells, tubule formation assay

## Abstract

The liver fluke *Opisthorchis viverrini* is a food-borne, zoonotic pathogen endemic to Thailand and adjacent countries in Southeast Asia. The adult developmental stage of the *O. viverrini* parasite excretes and secretes numerous proteins within the biliary tract including the gall bladder. Lesions caused by the feeding activities of the liver fluke represent wounds that undergo protracted cycles of healing and re-injury during chronic infection, which can last for decades. Components of the excretory/secretory (ES) complement released by the worms capably drive proliferation of bile duct epithelial cells and are implicated in establishing the oncogenic milieu that leads to bile duct cancer, cholangiocarcinoma. An ES protein, the secreted granulin-like growth factor termed *Ov*-GRN-1, accelerates wound resolution in mice and *in vitro*. To investigate angiogenesis (blood vessel development) that may contribute to wound healing promoted by liver fluke granulin and, by implication, to carcinogenesis during chronic opisthorchiasis, we employed an *in vitro* tubule formation assay (TFA) where human umbilical vein endothelial cells were grown on gelled basement matrix. Ten and 40 nM *Ov*-GRN-1 significantly stimulated angiogenesis as monitored by cellular proliferation and by TFA in real time. This demonstration of potent angiogenic property of *Ov*-GRN-1 bolsters earlier reports on the therapeutic potential for chronic non-healing wounds of diabetics, tobacco users, and the elderly and, in addition, showcases another of the hallmark of cancer characteristic of this carcinogenic liver fluke.

## Introduction

Infection with the fish-borne liver flukes *Opisthorchis viverrini, Opisthorchis felineus*, and *Clonorchis sinensis* remains a major public health problem in East Asia and Eurasia with >40 million cases. *O. viverrini* is endemic in regions of Thailand, Cambodia, Lao PDR, and Vietnam (1), and opisthorchiasis has been extensively studied in Thailand where ~8 million people are infected, calculated from nation-wide prevalence of 9.4% for the Thai population in 2001 (2, 3). Eating undercooked fish infected with the metacercariae stage of the fluke leads to infection in humans and other mammals, such as cats and dogs (1). The metacercarial stage of the parasite excysts in the duodenum and the juvenile fluke migrates into the bile ducts of the liver and matures over a month into an adult fluke, which grazes on biliary epithelia. The parasites are long lived, and often persist in the biliary tree for decades (1, 3). Fluke eggs are shed into the bile and exit with the fecal stream (1). Eggs that enter fresh water ecosystems can be ingested by the gastropod snail *Bithynia siamensis* (2, 4). The parasite develops within the snail, in turn releasing cercariae that seek out and penetrate the skin of a cyprinid fish, which encyst in the fish as a metacercaria, the infective stage for humans and other definitive host species (1).

Infection causes hepatobiliary disease, including cholangitis and periductal fibrosis (1). More problematically, both experimental and epidemiological evidence strongly implicates liver fluke infection in the etiology of cholangiocarcinoma (CCA), commonly known as bile duct cancer—one of the major liver cancer subtypes (1, 5). Up to 81% of liver cancers in the endemic Isaan region in north-eastern Thailand are CCA, which also suffers the world’s highest incidence of CCA—65 times the rate experienced in non-endemic regions (1, 5, 6). CCA is an adenocarcinoma that generally exhibits slow growth and which is diagnosed at advanced stage often with metastasis to distant sites due to proximity to lymphatic vessels (4). Unfortunately, prognosis is dismal at the advanced stage when the primary tumor is no longer amenable to liver resection. The mechanism(s) by which infection initiates genetic lesions that eventually culminate in CCA is unclear, but it likely involves biliary tract and systemic inflammation, inflammation associated endogenous and dietary nitrosation, and secretion of mitogens and other by the liver fluke.

One aspect of liver fluke infection that is potentially involved in malignant transformation is the excessive, unremitting wound healing in response to continued feeding by the parasites on bile duct tissue (2, 3, 5). We have shown that the granulin-like growth factor, *Ov*-GRN-1, secreted by the fluke is the dominant proliferative factor and is sufficient to drive wound healing (2, 7). A critical step in new tissue generation, including wound healing and tumor growth induced by the infection, is the stimulation of angiogenesis—the formation of new capillaries from pre-existing blood vessels or vasculogenic stem cells (8). New tissue requires angiogenesis to supply oxygen and nutrients, facilitate immune surveillance, and remove waste products (9). A complex interplay of growth factors and inhibitors regulates angiogenesis and imbalance can lead to disease (10). While angiogenesis does not itself initiate malignancy, it can promote tumor progression and metastasis (9, 11). Angiogenic cytokines, including fibroblast growth factor, vascular endothelial growth factor (VEGF), platelet-derived growth factor, and epidermal growth factor (9, 10), stimulate endothelial cells or precursors to proliferate and migrate, leading rapidly to new capillaries and blood vessel networks.

The tubule formation assay (TFA) provides an informative, convenient, rapid, and quantifiable approach to investigate angiogenesis (12). The TFA involves endothelial cell adhesion, migration, proteolysis, and tubule formation by the endothelial cells, which is initiated following seeding of the cells onto gelled basement matrix, the natural substrate of endothelial cell progenitors. The endothelial cells form capillary-like structures with a lumen (12). Recombinant *Ov*-GRN-1 (r*Ov*-GRN-1) induces angiogenesis (blood vessel growth) in quail embryos in the chorioallantoic membrane (CAM) assay (2). However, the angiogenic potential of liver fluke granulin on human cells has not been determined. Herein, we report potent angiogenic and mitogenic activity of *Ov*-GRN-1 at nanomolar concentration on primary human umbilical vein endothelial cells (HUVECs) using both a high-throughput TFA with automated ImageJ-based analyses and with the xCELLigence system real-time cell assay.

## Materials and Methods

### Ov-GRN-1 Recombinant Production

Purification of r*Ov*-GRN-1 was achieved using an AKTA10 purification system at 4°C (GE Healthcare) as previously described (2, 13). Briefly, BL21 *E. coli* bacterial pellet containing the r*Ov*-GRN-1 expression plasmid was lysed with three freeze/thaw cycles followed by sonication. The resulting insoluble pellet was solubilized in urea-containing nickel binding buffer [8 M urea/300 mM NaCl/50 mM imidazole/50 mM sodium phosphate pH 8 (Sigma)]. The 0.22-µM-filtered supernatant was passed over 2 × 5 ml Histrap IMAC nickel columns (GE Healthcare) and washed with increasing imidazole concentrations and eluted with 500-mM imidazole in binding buffer. Refolding of urea-denatured r*Ov*-GRN-1 was performed with 28 ml of G10 Sephadex resin on a XK16/20 column (GE Healthcare) with 7 ml applied at 100 µg/ml and eluted with 150-mM NaCl, 50-mM sodium phosphate, pH 6. A 120-ml Superdex 30 XK16/60 column (GE Healthcare) was used to fractionate r*Ov*-GRN-1 monomer eluting at a folded size equivalent to ~1 kDa. Protein concentration was determined by a combination of microplate Bradford assay (Bio-Rad) according to the manufacturer’s instructions and optical density at 280 nm.

### Human Umbilical Vein Endothelial Cells

Human umbilical vein endothelial cells pooled from donors (PromoCell, Heidelberg, Germany) were cultured in T75 tissue culture flasks in complete EGM-2 medium (PromoCell) at 37°C in a humidified atmosphere in 5% CO_2_ in air. HUVECs were grown to ~80% confluence, after which the culture was trypsinized using the PromoCell Detach Kit (PromoCell). The cells investigated by tubule formation assay (TFA) were used from the third, fourth, or fifth passage only, with HUVEC passaged to ~80% confluence within 24 h of the TFA, as described (14).

### Proliferation xCELLigence Assay

Cells were seeded at 5,000 cells per well in 200 µl of complete media (above) in E-plates (ACEA Biosciences, San Diego, CA, USA) and grown overnight while monitored with an xCELLigence DP system (ACEA Biosciences) which monitors cellular events in real time by measuring electrical impedance across interdigitated gold micro-electrodes integrated on the bottom of tissue culture plates. Cells were washed three times with PBS and replaced with 180 µl EGM-2 basal media (no growth factors or supplements) and incubated for a minimum of 6 h before further treatment. Treatments were prepared at 10 × concentrations and added to each well in a total volume of 20 µl. The xCELLigence DP recorded cell index readings every 15 min for 3 days after treatment. Cell index readings were normalized before treatment and cell proliferation ratios were determined from four biological replicates and represent the relative numbers of cells compared to control cells. A two-way ANOVA with Holm–Sidak’s multiple comparisons test was used to compare *Ov*-GRN-1 treatment to medium-alone control, with *P* ≤ 0.05 deemed significant.

### Tubule Formation Assay (TFA)

Growth factor-reduced Matrigel (Corning, Corning, NY, USA) was plated into a 96-well μ-angiogenesis plate (ibidi, Planegg, Germany) at 10 μl/well, and incubated at 37°C in 5% CO_2_ in air for 60 min as described (14). HUVECs were detached (above) and resuspended in complete endothelial cell growth medium 2 (EGM-2) (PromoCell), and seeded at 10,000 cells/well in medium supplemented with 10 µM sulforaphane (SFPH, Sigma) (negative control), 1.2 nM VEGF-165 (Novus Biologicals) (positive control), or 5, 10, 20, and 40 nM *Ov*-GRN-1. The ibidi plate was incubated for 12 h in a humidified atmosphere of 5% CO_2_ in air at 37°C in a microscope stage top incubator (OKOLAB, Pozzuoli, Naples, Italy). At intervals, photomicrographs of cells and nascent and developed tubules were collected using a Leica DMi8 automated platform microscope under bright field at 2.5 × magnification, and Leica LASX software (Leica).

### Analysis of Tubule Formation

Automated angiogenesis assessment was performed on TFA 490^2^ pixel images by ImageJ (NIH) with the phase-contrast Angiogenesis Analyzer plugin tool as described (12, 15). Settings used were as follows: 10 pixel minimum object size; 25 pixel minimum branch size; 2,500 pixel artifactual loop size; 25 pixel isolated element size threshold; 30 pixel master segment size threshold; with iteration number of 3. The four output metrics (mesh count, segment count, segment length, and junction count) were either plotted directly or as a percentage relative to the medium-alone blank treatment (treatment measure divided by medium-alone measure). A two-way ANOVA with Holm–Sidak’s multiple comparisons test was used to compare *Ov*-GRN-1 treatment against medium-alone blank control for the four metrics with *P* ≤ 0.05 deemed significant.

Combining the four metrics into a single evenly weighted variable was accomplished through the calculation of *Z* standardized scores that were based on population values (16). The formula below generates the *Z* score and represented the distance between the raw score and the population mean in units of the SD. Population values were estimated from 39 treatment replicates.

Z=(treatment metric value−metric population mean)/population standard deviation

The combined robust *Z* score (Z*) was generated for each replicate from the median *Z* score of the four metrics. Z* scores were plotted and *Ov*-GRN-1 treatments compared to medium-alone blank control using one-way ANOVA with Holm–Sidak’s multiple comparisons test, *P* ≤ 0.05 was considered to be statistically significant.

## Results

**Figure 1:**
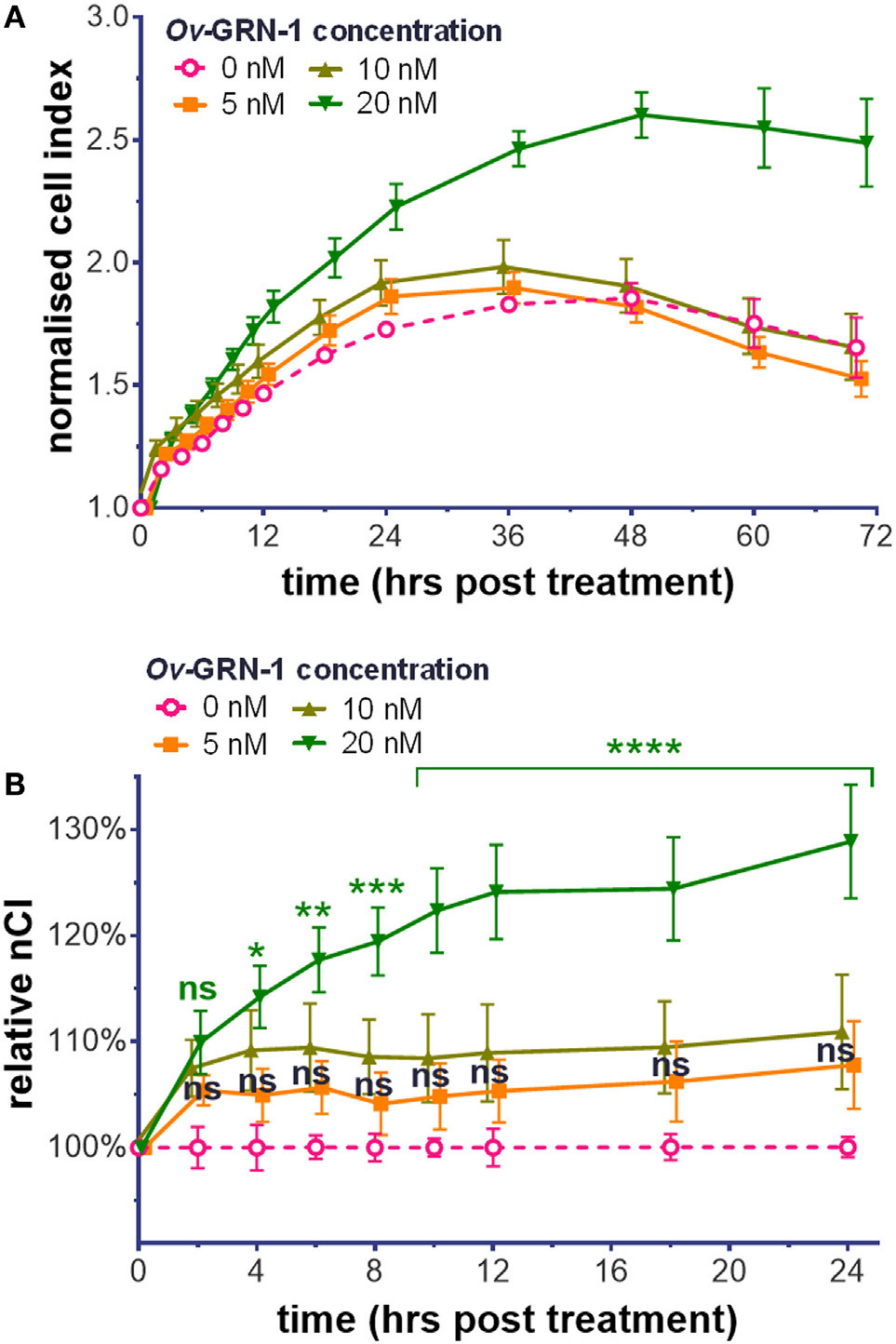
Cell proliferation induction by *Ov*-GRN-1 with human umbilical vein endothelial cells (HUVECs). **(A)** Normalized cell index (nCI) output of the xCELLigence system of treatments of 5–20 nM *Ov*-GRN-1 on HUVECs cells over 70 h. **(B)** nCI relative to medium only negative controls (0 nM *Ov*-GRN-1) from **(A)** is plotted to highlight the first 24 h after treatment. RM two-way ANOVA with Holm–Sidak’s multiple comparison test of *Ov*-GRN-1 treatment vs medium only control at each time point: ns = not significant; *P < 0.05; **P < 0.01; ***P < 0.001; ****P < 0.0001. Data points are average values of four biological replicates and have been nudged ±0.2–1 h to ensure that SE bars are visible.

To assess the influence of *Ov*-GRN-1 on HUVEC compared with other cell types, we analyzed HUVEC cellular proliferation and migration with the xCELLigence system. Treatment with *Ov*-GRN-1 induces proliferation in a range of cell types (2, 4, 17). Exposure of HUVEC to 5–20 nM *Ov*-GRN-1 showed a similar response (Figure 1). As the angiogenic assay with HUVECs would be assessed at 12 h, for proliferation, we focused on the first 24 h and noted minor (5–11%) non-significant increase above values of negative controls from 5 to 10 nM *Ov*-GRN-1 (Figure 1B). Twenty nanomolars were sufficient to induce a significant 14% increase in cell index from 4 h (*P < 0.05) that had expanded to 29% by 24 h (****P < 0.0001), indicative of proliferation (Figure 1B). Cell migration has also been reported with bile duct cells from *Ov*-GRN-1 treatment (2, 3). In contrast to induction of proliferation, our HUVEC tests with 10–100 nM *Ov*-GRN-1 using CIM e-plates (cell invasion and migration xCELLigence plates) did not reveal an increase in migration compared to controls (data not shown).

**Figure 2:**
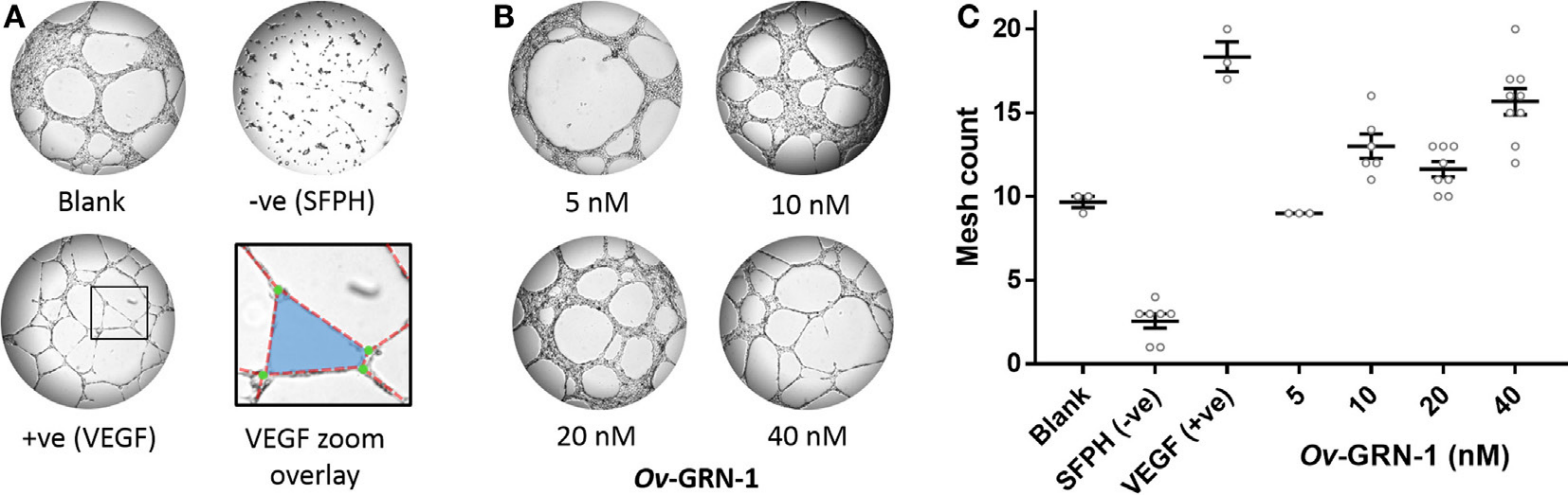
Angiogenic screening with primary human umbilical vein endothelial cells. Cells are seeded and treatments applied at time zero. Images are taken at 12 h and assessed in ImageJ for angiogenic properties. **(A)** Representative images depict the control treatments at 50× magnification: medium only; anti-angiogenic negative control [10 µM sulfurophane (SFPH)]; pro-angiogenic positive control [1.2 nM vascular endothelial growth factor (VEGF)]. The VEGF zoom overlay shows the boxed VEGF section magnified with angiogenic features overlaid: A mesh (blue) is bordered by four tubule segments (red dotted lines) that join at four branching junctions (green circles). **(B)** Representative images of 5–40 nM *Ov*-GRN-1 treatments at 50× magnification. **(C)** Automated mesh count quantitation of control and *Ov*-GRN-1 treatments represent network complexity. The mean is marked as a horizontal bar with SE bars from 3 to 9 biological replicates plotted as circles.

The TFA angiogenesis was used to assess the ability of r*Ov*-GRN-1 to induce capillary-like tubule structures from endothelial cells. The three main characteristics considered for angiogenic stimulation in the TFA are highlighted in Figure 2A—tubule “segments” branch at “junctions” and combine to enclose a “mesh.” Ten (10) micromolar SFPH negative control revealed an ablated tubule network, as reported in other studies (18), whereas positive control VEGF at 1.2 nM strongly stimulated a network of tubules compared to the medium only group (19) (Figure 2A).

**Figure 3:**
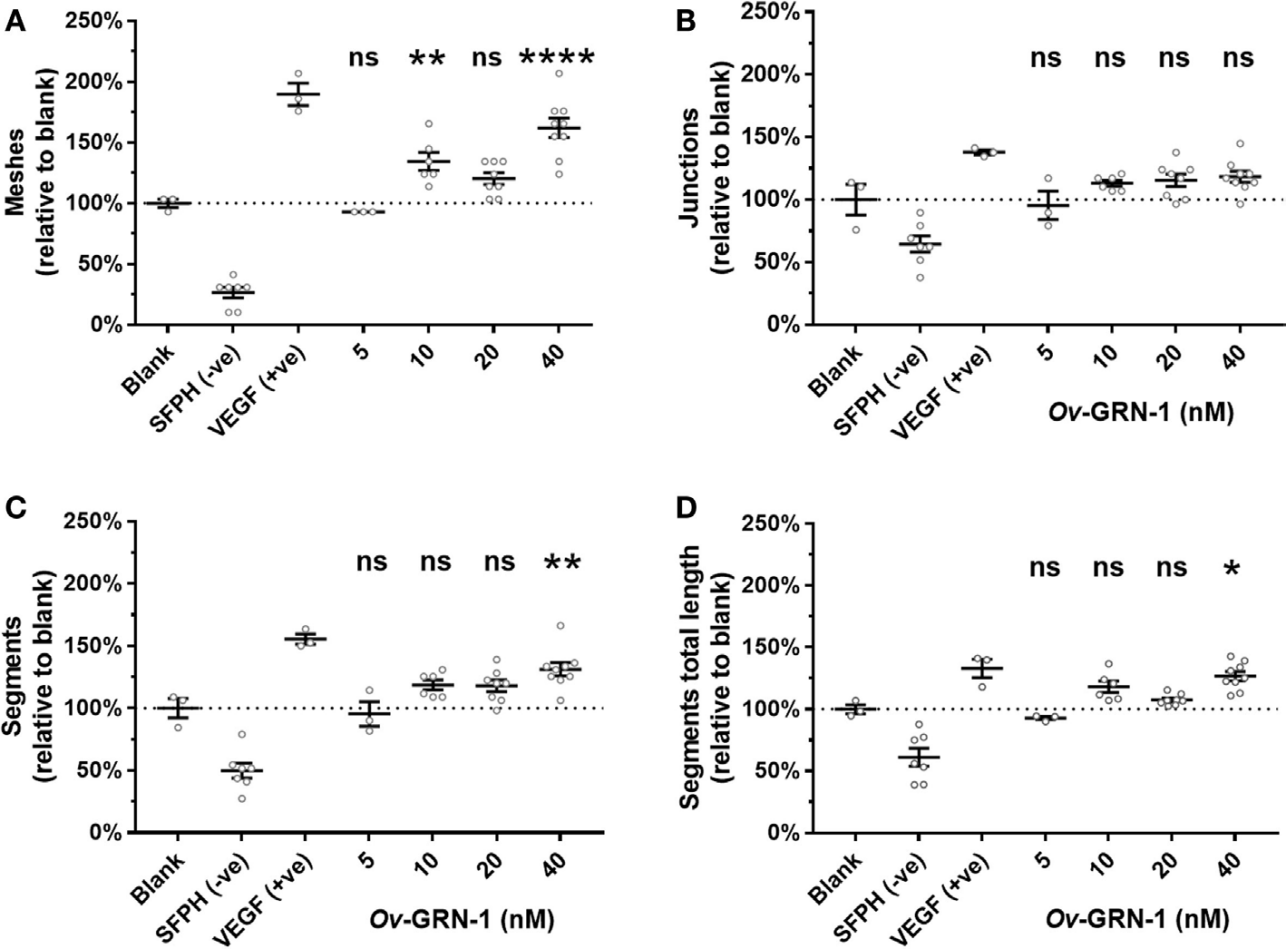
Angiogenic metrics from *Ov*-GRN-1 treatments. ImageJ was used to quantify 12-h time point images for various characteristics of the tubule networks. Treatments consist of the blank control (medium only); anti-angiogenic negative control [10 µM sulforaphane (SFPH)]; pro-angiogenic positive control [1.2 nM vascular endothelial growth factor (VEGF)]; and 5–40 nM *Ov*-GRN-1 treatments and are plotted relative to the blank (medium only) control treatment. **(A)** Topological features representing an increase in complexity of the tubule network are measured by the relative number of meshes and **(B)** branching junctions. **(C)** Dimensional descriptors of the networked tubules include the number of tubule segments and **(D)** the total combined length of the tubule segments. All panels: two-way ANOVA with Holm–Sidak’s multiple comparisons test to compare *Ov*-GRN-1 treatment to medium only blank control: ns = not significant; *P < 0.05; **P < 0.01; ****P < 0.0001. The mean is marked as a horizontal bar with SE bars from 3 to 9 biological replicates plotted as circles. Dotted line crosses the medium only blank 100% value.

The automated ImageJ angiogenesis plugin analysis measures both topological features that represent network complexity (meshes and branching junction points) and morphometric descriptors that are dimensional and considered the principal angiogenic *in vitro* response (tubule segment number and total tubule segment length) (8). *Ov*-GRN-1 treatments show a concentration dependent increasingly complex tubule mesh network (Figure 2). The mesh count raw data (Figure 2C) show that 40 nM *Ov*-GRN-1 increased the mesh count from the medium only control of 9.7–15.7 meshes—a highly significant (*P* < 0.0001) relative increase over the blank control of 62% (Figure 3A). In addition, 10 and 20 nM *Ov*-GRN-1 increased the mesh count to a lesser degree—35% increase (*P* < 0.01) and a 20% increase (non-significant), respectively. While the number of branching junctions (Figure 3B) increased slightly (13–18%) with 10–40 nM *Ov*-GRN-1 treatments, none of these changes were significantly different to the medium only control. The dimensional measures both showed slight increases of 8–18% with 10–20 nM but only significant increases from 40 nM *Ov*-GRN-1 treatment (Figures 3C,D). The number of tubule segments increased by 31% (*P* < 0.01) and the associated total length of the tubule segments increased by 27% (*P* < 0.05) to the medium only control. The 5 nM *Ov*-GRN-1 treatment showed almost no difference (non-significant minor reductions of 5–8%) for all four metrics relative to medium only controls.

**Figure 4:**
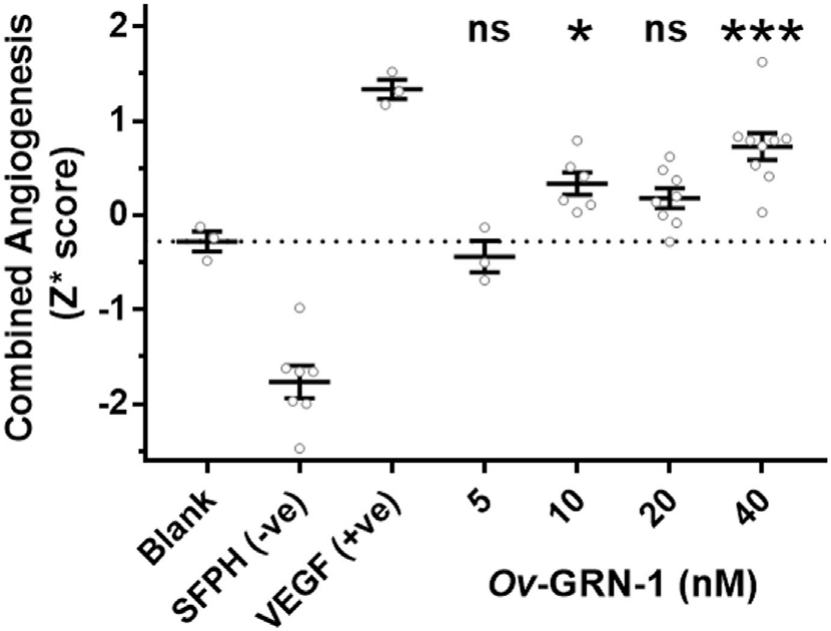
Angiogenic induction from *Ov*-GRN-1 treatments. Converting the four angiogenic metrics (meshes, junctions, segments, and segment length) to dimensionless *Z*-scores allows a pooled median value for each replicate (Z* score) to be generated. This enables the total angiogenic effect to be compared between groups. One-way ANOVA with Holm–Sidak’s multiple comparisons test to compare *Ov*-GRN-1 treatment to medium only blank control: ns = not significant; *P < 0.05; **P < 0.01; ***P < 0.001. The mean is marked as a horizontal bar with SE bars from 3 to 9 biological replicates plotted as circles. Dotted line crosses the medium only blank −0.28 Z* score.

To assess the angiogenic potential of *Ov*-GRN-1 treatments the four metrics were converted to *Z*-scores and combined into a single median robust *Z* score (Z*) for each replicate (Figure 4). The Z* score incorporates differences from the total population mean and population variation. This dimensionless variable is a useful equally weighted mechanism to combine metrics with different means and variations into a single composite variable for statistical comparisons. Comparing the Z* scores to the medium only controls (Figure 4) showed 10–40 nM *Ov*-GRN-1 induced increased angiogenesis but only 10 and 40 nM showed significant increases (*P* < 0.05 and *P* < 0.001, respectively). As noted with the four separate metrics, 5 nM *Ov*-GRN-1 did not stimulate angiogenesis.

## Discussion

Angiogenesis describes the growth of new vasculature and is a process that has been the subject of significant historical interest due to its involvement in a spectrum of diseases (10, 20, 21). During development new blood vessels are essential and angiogenesis is rapid (9, 10). In the fully developed adult, as part of physiologic processes such as wound healing, angiogenesis is turned on, but only transiently (9, 10). Over a century ago, studies demonstrated that the mechanisms of tumor growth and spread are intimately linked to the formation of new blood vessels from pre-existing larger blood vessels (10). Subsequently, it was determined that as this process occurs, the “angiogenic switch” is almost always activated and remains on, causing normally quiescent vasculature to sustain expanding neoplastic growths (9).

Molecular angiogenic studies have focused on VEGF, as many angiogenic factors induce signals that result in blood vessel signaling that ultimately rely on VEGF (10, 21, 22). Recent reports focus on angiogenic signaling *via* VEGF-independent mechanisms and associated problems with VEGF inhibitors for cancer treatments (23–26). One such alternate pathway that is not blocked by VEGF inhibitors is driven by human progranulin, a growth factor comprised of 7.5 granulin active domains (24, 27). The individual human granulin units are difficult to produce actively (28) but *Ov*-GRN-1 is produced by liver flukes as a single unit, and is bioactive when purified from denatured bacterial recombinant expression systems and refolded (17). Previously, we have shown that *Ov*-GRN-1 is a potent growth factor that stimulates human cell proliferation, migration, avian angiogenesis, and ultimately mouse wound healing (3, 4, 7, 17). Ongoing research suggests the potential for beneficial therapies emanating from pathophysiological investigation of granulins, including anti-tumor action and promotion of wound healing (7, 29, 30).

The avian CAM assay is an informative *in vivo* model of angiogenesis (31). The CAM assay is based around blood vessel induction in avian embryos and results may not traverse phylogenetic boundaries and apply to mammalian systems. With this consideration ensuring the parasite growth factor *Ov*-GRN-1 stimulated human angiogenesis is a useful step toward developing therapeutics (2, 7, 10, 32). Herein, we focused on angiogenesis screening using HUVEC and the tubule formation assay (TFA) with automated imaging in real time (14). The TFA involves a range of the angiogenic processes that includes endothelial cell adhesion, migration, protease activity, and tubule formation (14). The advantages of the TFA to study angiogenesis are the ease of set up, short culture period, the ability to produce quantitative data, and its ease of adaptation toward high-throughput analysis. The automated TFA analysis allows comparison of both topological features that represent network complexity and morphometric dimensional descriptors that are considered the principal angiogenic *in vitro* response (8). This depth of analysis combined with the high-throughput capability allows a greater range of treatments to be tested than the labor-intensive and subjectively scored CAM assay.

Human liver epithelial cells (H69 bile duct cell line) hyper-proliferates at *Ov*-GRN-1 concentrations of 12 nM and above (7). Although the cell types were different, we note that *Ov*-GRN-1 is able to stimulate significant HUVEC proliferation and angiogenic stimulation at similar concentrations of at least 20 nM and 10 nM, respectively (Figures 1 and 4). Sample limitations restricted the *Ov*-GRN-1 TFA maximum concentration to 40 nM, but as the trend appeared to be dose dependent we predict that higher concentration would induce further angiogenesis, and plan to assess in the future. Mesh counts showed the most marked rise with a 62% highly significant increase (*P* < 0.0001) with 40 nM *Ov*-GRN-1, whereas the junction counts trended up (13–18%) but did not reach statistical significance (Figure 3). Whether the outcome related to the *Ov*-GRN-1 mechanism is not clear, but these phenomena may be an artifact of the TFA mechanics. A junction is the end point of at least three segments, and more may radiate from the connecting junction. Increased junction branching was evident with 40 nM *Ov*-GRN-1, a small increase in junctions (18%) but about double the increase in tubule segments (31%) was observed. The increase in segments can in turn enhance the mesh count, as a single segment tubule can grow across a mesh and split it into two smaller meshes; our estimated mesh numbers increased 62%. To explore this junction/mesh outcome further and delve into the angiogenic activity of *Ov*-GRN-1, we plan to test other aspects of angiogenesis, including apoptosis, and to employ three-dimensional cell culture systems that more closely reflect the *in vivo* situation (19, 33). Furthermore, future studies will explore the *Ov*-GRN-1 proliferative and signaling responses of HUVECs to compare with our previous work (2) that showed the transcriptional responses of *Ov*-GRN-1 treatment on the H69 bile duct cell line. The most relevant to *Ov*-GRN-1 transcriptional response to angiogenesis we observed in the H69 cells was the stimulation of CXCL1, 2, 5, and 8—chemokines that signal through CXCR2, a receptor with complex signaling interactions but often resulting in angiogenesis among other outcomes such as inflammation (22, 34).

Whereas the origin of helminth and host cell communication is unknown, the process likely evolved to facilitate parasitism (35). Metabolites participating in communication signaling may, however, contribute to carcinogenesis (1, 35). The close homology between liver fluke and human granulins may enable secreted *Ov*-GRN-1 to activate signaling pathways that promote angiogenesis and wound repair of bile ducts damaged by the activities of the parasite (4). The stimulation of wound healing potentially evolved for successful parasitism and a concomitant productive host–parasite relationship (2, 7). Other helminth parasites release mediators that promote angiogenesis, and from an evolutionary perspective, *Opisthorchis*-induced neovascularization may be beneficial to parasitism (35, 36). However, given that angiogenesis represents a hallmark of cancer, the angiogenic potency of *Ov*-GRN-1 may also contribute to liver fluke infection-induced malignancy (2, 30, 35).

The current findings confirmed potent angiogenic signaling of liver fluke with nanomolar levels of granulin and expand upon previous reports using an *in vivo* CAM assay (2). Liver fluke granulin likely contributes to the carcinogenicity of liver fluke infection in the human biliary tract (2, 35, 36). On the other hand, it also holds marked potential as a therapeutic wound-healing agent and as a vaccine against the liver fluke infection-induced cancer (2, 7, 35).

## Author Contributions

Study conception: PB, MS, and AL; performed experiments: BH, SK, and VM; data analysis: AP, MS and BH; data presentation: MS; manuscript preparation: MS, BH, SK, and PB; manuscript editing: MS, AL, BH, SK, VM, and PB; Supervision: AP, PB and MS, and Funding acquisition: PB and AL.

## Conflict of Interest Statement

The authors declare that the research was conducted in the absence of any commercial or financial relationships that could be construed as a potential conflict of interest.

## References

[1] Smout MJ Sripa B Laha T Mulvenna J Gasser RB Young ND Infection with the carcinogenic human liver fluke, *Opisthorchis viverrini*. Mol Biosyst (2011) 7(5):1367–75. 10.1039/c0mb00295j 21311794 PMC3739706

[2] Smout MJ Sotillo J Laha T Papatpremsiri A Rinaldi G Pimenta RN Carcinogenic parasite secretes growth factor that accelerates wound healing and potentially promotes neoplasia. PLoS Pathog (2015) 11(10):e1005209. 10.1371/journal.ppat.1005209 26485648 PMC4618121

[3] Papatpremsiri A Smout MJ Loukas A Brindley PJ Sripa B Laha T. Suppression of *Ov-grn-1* encoding granulin of *Opisthorchis viverrini* inhibits proliferation of biliary epithelial cells. Exp Parasitol (2015) 148:17–23. 10.1016/j.exppara.2014.11.004 25450776 PMC4277937

[4] Smout MJ Laha T Mulvenna J Sripa B Suttiprapa S Jones A A granulin-like growth factor secreted by the carcinogenic liver fluke, *Opisthorchis viverrini*, promotes proliferation of host cells. PLoS Pathog (2009) 5(10):e1000611. 10.1371/journal.ppat.1000611 19816559 PMC2749447

[5] Sripa B Brindley PJ Mulvenna J Laha T Smout MJ Mairiang E The tumorigenic liver fluke *Opisthorchis viverrini* – multiple pathways to cancer. Trends Parasitol (2012) 28(10):395–407. 10.1016/j.pt.2012.07.006 22947297 PMC3682777

[6] Sripa B Kaewkes S Sithithaworn P Mairiang E Laha T Smout M Liver fluke induces cholangiocarcinoma. PLoS Med (2007) 4(7):e201 10.1371/journal.pmed.0040201 17622191 PMC1913093

[7] Bansal PS Smout MJ Wilson D Cobos Caceres C Dastpeyman M Sotillo J Development of a potent wound healing agent based on the liver fluke granulin structural fold. J Med Chem (2017) 60(10):4258–66. 10.1021/acs.jmedchem.7b00047 28425707 PMC12212975

[8] Boizeau ML Fons P Cousseins L Desjobert J Sibrac D Michaux C Automated image analysis of in vitro angiogenesis assay. J Lab Autom (2013) 18(5):411–5. 10.1177/2211068213495204 23813914

[9] Chung AS Lee J Ferrara N. Targeting the tumour vasculature: insights from physiological angiogenesis. Nat Rev Cancer (2010) 10(7):505–14. 10.1038/nrc2868 20574450

[10] Chung AS Ferrara N. Developmental and pathological angiogenesis. Annu Rev Cell Dev Biol (2011) 27:563–84. 10.1146/annurev-cellbio-092910-154002 21756109

[11] Cimpean AM Ribatti D Raica M. A brief history of angiogenesis assays. Int J Dev Biol (2011) 55(4–5):377–82. 10.1387/ijdb.103215ac 21858762

[12] DeCicco-Skinner KL Henry GH Cataisson C Tabib T Gwilliam JC Watson NJ Endothelial cell tube formation assay for the in vitro study of angiogenesis. J Vis Exp (2014) 91:e51312. 10.3791/51312 25225985 PMC4540586

[13] Jin AH Dekan Z Smout MJ Wilson D Dutertre S Vetter I Conotoxin Phi-MiXXVIIA from the superfamily G2 employs a novel cysteine framework that mimics granulin and displays anti-apoptotic activity. Angew Chem Int Ed Engl (2017) 56(47):14973–6. 10.1002/anie.201708927 28984021 PMC12221324

[14] Arnaoutova I Kleinman HK. In vitro angiogenesis: endothelial cell tube formation on gelled basement membrane extract. Nat Protoc (2010) 5(4):628–35. 10.1038/nprot.2010.6 20224563

[15] Carpentier G Angiogenesis analyzer. Image J News (2012). Available from: http://image.bio.methods.free.fr/ImageJ/?Angiogenesis-Analyzer-for-ImageJ

[16] Zhang XD Ferrer M Espeseth AS Marine SD Stec EM Crackower MA The use of strictly standardized mean difference for hit selection in primary RNA interference high-throughput screening experiments. J Biomol Screen (2007) 12(4):497–509. 10.1177/1087057107300646 17435171

[17] Smout MJ Mulvenna JP Jones MK Loukas A. Expression, refolding and purification of Ov-GRN-1, a granulin-like growth factor from the carcinogenic liver fluke, that causes proliferation of mammalian host cells. Protein Expr Purif (2011) 79(2):263–70. 10.1016/j.pep.2011.06.018 21757010

[18] Bertl E Bartsch H Gerhauser C. Inhibition of angiogenesis and endothelial cell functions are novel sulforaphane-mediated mechanisms in chemoprevention. Mol Cancer Ther (2006) 5(3):575–85. 10.1158/1535-7163.MCT-05-0324 16546971

[19] Arutyunyan I Fatkhudinov T Kananykhina E Usman N Elchaninov A Makarov A Role of VEGF-A in angiogenesis promoted by umbilical cord-derived mesenchymal stromal/stem cells: in vitro study. Stem Cell Res Ther (2016) 7:46. 10.1186/s13287-016-0305-4 27001300 PMC4802928

[20] Salvi V Vermi W Gianello V Lonardi S Gagliostro V Naldini A Dendritic cell-derived VEGF-A plays a role in inflammatory angiogenesis of human secondary lymphoid organs and is driven by the coordinated activation of multiple transcription factors. Oncotarget (2016) 7(26):39256–69. 10.18632/oncotarget.9684 27256980 PMC5129930

[21] Morgan C Nigam Y. Naturally derived factors and their role in the promotion of angiogenesis for the healing of chronic wounds. Angiogenesis (2013) 16(3):493–502. 10.1007/s10456-013-9341-1 23417553

[22] Rosenkilde MM Schwartz TW The chemokine system – a major regulator of angiogenesis in health and disease. APMIS (2004) 112(7–8):481–95. 10.1111/j.1600-0463.2004.apm11207-0808.x 15563311

[23] Molina F Del Moral ML Peinado MA Rus A. Angiogenesis is VEGF-independent in the aged striatum of male rats exposed to acute hypoxia. Biogerontology (2017) 18(5):759–68. 10.1007/s10522-017-9709-5 28501895

[24] Eguchi R Nakano T Wakabayashi I. Progranulin and granulin-like protein as novel VEGF-independent angiogenic factors derived from human mesothelioma cells. Oncogene (2017) 36(5):714–22. 10.1038/onc.2016.226 27345409

[25] Petrillo M Borriello M Fuoco G Legge F Iannone V Ferrandina G. Novel VEGF-independent strategies targeting tumor vasculature: clinical aspects. Curr Pharm Des (2012) 18(19):2702–12. 10.2174/138161212800626184 22390758

[26] Dai J Wan S Zhou F Myers RE Guo X Li B Genetic polymorphism in a VEGF-independent angiogenesis gene ANGPT1 and overall survival of colorectal cancer patients after surgical resection. PLoS One (2012) 7(4):e34758. 10.1371/journal.pone.0034758 22496856 PMC3319640

[27] Yang D Wang LL Dong TT Shen YH Guo XS Liu CY Progranulin promotes colorectal cancer proliferation and angiogenesis through TNFR2/Akt and ERK signaling pathways. Am J Cancer Res (2015) 5(10):3085–97. 26693061 PMC4656732

[28] Tolkatchev D Malik S Vinogradova A Wang P Chen Z Xu P Structure dissection of human progranulin identifies well-folded granulin/epithelin modules with unique functional activities. Protein Sci (2008) 17(4):711–24. 10.1110/ps.073295308 18359860 PMC2271164

[29] Ho JC Ip YC Cheung ST Lee YT Chan KF Wong SY Granulin-epithelin precursor as a therapeutic target for hepatocellular carcinoma. Hepatology (2008) 47(5):1524–32. 10.1002/hep.22191 18393387

[30] Osherov N Ben-Ami R Modulation of host angiogenesis as a microbial survival strategy and therapeutic target. PLoS Pathog (2016) 12(4):e1005479 10.1371/journal.ppat.1005838 27078259 PMC4831739

[31] Ribatti D. Chick embryo chorioallantoic membrane as a useful tool to study angiogenesis. Int Rev Cell Mol Biol (2008) 270:181–224. 10.1016/S1937-6448(08)01405-6 19081537

[32] Chan LY Gunasekera S Henriques ST Worth NF Le SJ Clark RJ Engineering pro-angiogenic peptides using stable, disulfide-rich cyclic scaffolds. Blood (2011) 118(25):6709–17. 10.1182/blood-2011-06-359141 22039263

[33] Aranda E Owen GI. A semi-quantitative assay to screen for angiogenic compounds and compounds with angiogenic potential using the EA.hy926 endothelial cell line. Biol Res (2009) 42(3):377–89. 10.4067/S0716-97602009000300012 19915746

[34] Raghuwanshi SK Su Y Singh V Haynes K Richmond A Richardson RM. The chemokine receptors CXCR1 and CXCR2 couple to distinct G protein-coupled receptor kinases to mediate and regulate leukocyte functions. J Immunol (2012) 189(6):2824–32. 10.4049/jimmunol.1201114 22869904 PMC3436986

[35] Brindley PJ Loukas A Helminth infection-induced malignancy. PLoS Pathog (2017) 13(7):e1006393 10.1371/journal.ppat.1006393 28750101 PMC5531427

[36] Dennis RD Schubert U Bauer C. Angiogenesis and parasitic helminth-associated neovascularization. Parasitology (2011) 138(4):426–39. 10.1017/S0031182010001642 21232174

